# Biogeochemical implications of the ubiquitous colonization of marine habitats and redox gradients by *Marinobacter* species

**DOI:** 10.3389/fmicb.2013.00136

**Published:** 2013-05-22

**Authors:** Kim M. Handley, Jonathan R. Lloyd

**Affiliations:** ^1^Searle Chemistry Laboratory, Computation Institute, University of ChicagoChicago, IL, USA; ^2^Computing, Environment and Life Sciences, Argonne National LaboratoryChicago, IL, USA; ^3^School of Earth, Atmospheric, and Environmental Sciences, University of ManchesterManchester, UK

**Keywords:** *Marinobacter*, marine, hydrothermal, biogeochemical cycling, hydrocarbon, iron, arsenic, opportunistic

## Abstract

The *Marinobacter* genus comprises widespread marine bacteria, found in localities as diverse as the deep ocean, coastal seawater and sediment, hydrothermal settings, oceanic basalt, sea-ice, sand, solar salterns, and oil fields. Terrestrial sources include saline soil and wine-barrel-decalcification wastewater. The genus was designated in 1992 for the Gram-negative, hydrocarbon-degrading bacterium *Marinobacter hydrocarbonoclasticus*. Since then, a further 31 type strains have been designated. Nonetheless, the metabolic range of many *Marinobacter* species remains largely unexplored. Most species have been classified as aerobic heterotrophs, and assessed for limited anaerobic pathways (fermentation or nitrate reduction), whereas studies of low-temperature hydrothermal sediments, basalt at oceanic spreading centers, and phytoplankton have identified species that possess a respiratory repertoire with significant biogeochemical implications. Notable physiological traits include nitrate-dependent Fe(II)-oxidation, arsenic and fumarate redox cycling, and Mn(II) oxidation. There is also evidence for Fe(III) reduction, and metal(loid) detoxification. Considering the ubiquity and metabolic capabilities of the genus, *Marinobacter* species may perform an important and underestimated role in the biogeochemical cycling of organics and metals in varied marine habitats, and spanning aerobic-to-anoxic redox gradients.

## Introduction

Marine habitats are host to a diverse range of substrates and physicochemical regimes. Among these, hydrothermal features attract particular interest owing to ore-grade concentrations of metals, physicochemical extremes, and the presence of chemolithoautotrophic macrofauna and microbiota. Bacteria and Archaea occupying marine habitats have a substantial physical presence. There are an estimated 3.9 × 10^30^ prokaryotic cells in the open ocean and unconsolidated marine sediments, comprising ~3.1 × 10^11^ tonnes of carbon (Whitman et al., 1998). This is slightly more than the estimated carbon content of terrestrial prokaryotes, and just under half of that in all plant life. Prokaryotes can contribute significantly to marine ecosystem functioning and biogeochemical cycles (Jørgensen, 2006), owing to their prevalence and enormous capacity for transforming their environments through metabolism of organic and inorganic matter (Gadd, 2010). Yet much of the marine microbial biomass remains unexplored, and there is still much to learn about heterotrophic and autotrophic bacterial functioning in the ocean (e.g., Moran et al., 2004; Emerson et al., 2010; Holden et al., 2012).

*Marinobacter* is a heterotrophic, and in some instances mixotrophic (Dhillon et al., 2005; Handley et al., 2009a,b), metabolically flexible genus found in an exceptionally wide range of marine and saline terrestrial settings, including various low-temperature hydrothermal environments (Table 1). The genus comprises Gram-negative *Gammaproteobacteria* within the *Alteromonadales* order. All known species are motile with polar flagella (excluding *M. goseongensis*, Roh et al., 2008), slightly to moderately halophilic (cf. DasSarma and Arora, 2012), aerobic heterotrophs (Table 2). However, few are confirmed strict aerobes, and several are facultative anaerobes (Table 1). All are rod-shaped, with the exception of the ellipsoidal *M. Segnicrescens* (Guo et al., 2007), and most are neutrophilic, except the slightly alkaliphilic *M. alkaliphilus*, which grows optimally at pH 8.5–9.0 (Takai et al., 2005; also see Al-Awadhi et al., 2007; Table 2). Although most species are mesophilic, many are psychrotolerant (also known as psychrotrophic) and capable of growth down to ~4°C (Table 2; Moyer and Morita, 2007). A couple of other species are either psychrophilic (with a growth optimum near 15°C) or thermotolerant (with growth up to 50°C and an optimum of 45°C). This phenotypic versatility contributes to the ubiquity of this genus, and its ability to occupy diverse physicochemical regimes.

**Table 1:** ***Marinobacter* species metabolism and isolation source**.

***Marinobacter* species**	**HC**	**CH^b^**	**NO^−^_3_**	**Gluc ferm**	**AA**	**Anaer**	**Env**	**Isolation source**
*hydrocarbonoclasticus*^1^^,^^a^	Y	N	resp	N	Y	Y	–	Oil-polluted sediment; Gulf of Fos; Mediterranean coast; France
*aquaeolei*^2^	Y	N	resp	N	Y	Y	–	Oil-producing well-head; offshore platform; Vietnam
*excellens*^3^^,^^a^	–	Y	resp	Y	Y	Y	12°C	Radionuclide-polluted sediment; 0.5 m depth; Chazhma Bay; Sea of Japan; Russia
*lipolyticus*^4^^,^^a^	–	Y	N	–	N	st-aer	–	Saline soil; seaside city of Cádiz; Spain
*squalenivorans*^5^	Y	–	resp	N	Y	Y	–	Oil-contaminated coastal sediment; Carteau Cove; Gulf of Fos; France
*lutaoensis*^6^^,^^a^	–	Y	N	N	Y	st-aer	43°C	Coastal hot spring water; Lutao; Taiwan
*litoralis*^7^^,^^a^	–	–	Y	N	N	N	–	Sea water; Jungdongjin beach; East Sea; Korea
*flavimaris*^8^^,^^a^	–	Y	Y	–	N	Y	–	Sea water; Daepo Beach; Yellow Sea; Korea
*daepoensis*^8^^,^^a^	–	–	N	–	N	Y	–	Sea water; Daepo Beach; Yellow Sea; Korea
*bryozoorum*^9^^,^^a^	–	Y	Y	–	Y	Y	–	Sediment; Bearing Sea; Russia
*sediminum*^9^^,^^a^	–	Y	–	–	Y	–	–	Sediment; Peter the Great Bay; Sea of Japan; Russia
*maritimus*^10^^,^^a^	Y	Y	–	–	Y	–	–	Sea water; 110 km SW of subantarctic Kerguelen islands
*alkaliphilus*^11^	Y	Y	Y	–	Y	Y	1.7–1.9°C	Subseafloor alkaline serpentine mud; South Chamorro Seamount; Mariana Forearc
*algicola*^12^^,^^a^	Y	Y	resp	–	Y	Y	–	Dinoflagellate *Gymnodinium catenatum*; Yellow Sea; Korea
*koreensis*^13^^,^^a^	–	N	Y	N	Y	Y	–	Sea-shore sand at Homi Cape; Pohang; Korea
*vinifirmus*^14^^,^^a^	–	N	N	N	N	st-aer	–	Wine tank decalcification wastewater-evaporation pond. Location?
*salsuginis*^15^^,^^a^	Y	Y	resp	Y	Y	Y	–	Brine-seawater interface; Shaban Deep (a brine-filled deep); Red Sea
*gudaonensis*^16^^,^^17^^,^^a^	Y	Y	Y	–	Y	Y	–	Oil-polluted soil underlying wastewater from the coastal Shengli Oil field; China
*segnicrescens*^17^^,^^a^	N	Y	Y	–	N	Y	–	Benthic sediment; 1161 m depth; South China Sea
*salicampi*^18^^,^^a^	–	N	Y	–	–	Y	–	Sediment; marine solar saltern; Yellow Sea; Korea
*pelagius*^19^^,^^a^	–	N	Y	–	Y	–	–	Coastal seawater; Zhoushan Archipelago; China
*guineae*^20^^,^^a^	–	Y	resp	N	–	Y	–	Marine sediment; Deception Island; Antarctica
*psychrophilus*^21^^,^^a^	–	Y	Y	–	Y	–	Freezing	Sea-ice; Canadian Basin; Arctic Ocean
*mobilis; zhejiangensis*^22^^,^^a^	–	N	Y	–	Y	–	–	Sediment; Dayu Bay; East China Sea (Lat. 27.33, Long. 120.57)
*goseongensis*^23^^,^^a^	–	N	–	–	N	–	–	Coastal seawater; 100 m depth; East Sea of Korea
*santoriniensis*^24^^,^^a^	G	N	resp	N	–	Y	25°C	Ferruginous hydrothermal marine sediment; Santorini; Greece
*szutsaonensis*^25^^,^^a^	–	Y	Y	–	Y	–	16–17°C	Soil; Szutsao solar saltern; southern Taiwan
*lacisalsi*^26^^,^^a^	–	N	Y	–	N	–	–	Water; hypersaline lake; ~50 km inland; saline-wetland; Fuente de Piedra; Spain
*zhanjiangensis*^27^^,^^a^	–	Y	Y	–	N	–	–	Sea water; tidal flat, Naozhou Island; South China Sea
*oulmenensis*^28^^,^^a^	–	Y	N	N	Y	–	–	Brine; salt concentrator (input material?); ~60 km inland; Ain Oulmene; Algeria.
*daqiaonensis*^29^^,^^a^	–	Y	N	N	–	–	–	Sediment; Daqiao salt pond; Yellow Sea; east coast of China
*adhaerens*^30^^,^^a^	–	Y	N	–	Y	–	–	*Thalassiosira weissflogii* diatom aggregates; Wadden sea surface; Germany
*antarcticus*^31^^,^^a^	–	Y	Y	–	Y	–	–	Intertidal sandy sediment; Larsemann Hills; Antarctica
*xestospongiae*^32^^,^^a^	–	Y	Y	Y	Y	–	–	Coastal marine sponge; 8 m depth; Obhor Sharm; Red Sea; Saudi Arabia
Terrestrial strain MB^33^	–	–	–	–	–	Y	–	Cyanobacterial mat; saline lake; near the Red Sea
*manganoxydans*^34^	G	–	–	–	–	–	–	Heavy metal-rich sediment; hydrothermal vent; Indian Ocean (Lat. 25.32, Long. 70.04)
*Marinobacter*-like isolates^35^^,^^c^	–	N	–	–	–	Y	~4°C	Weathering metal sulfide rock and sediment; Main Endeavour/Middle Valley; JdFR
*Marinobacter*-like clones^36^^,^^d^	–	–	–	–	–	–	≥4°C	Metal sulfides rock and sediment; Main Endeavour/Middle Valley; JdFR
*Marinobacter* clones^37^^,^^e^	–	–	–	–	–	–	4°C	Relict 50 ka metal sulfide sediment; Alvin mound; TAG; Mid-Atlantic Ridge
*Marinobacter* isolates^38^^,^^f^	–	–	–	–	–	–	~2°C	Lateral hydrothermal plumes; Mothra vent field and Axial Seamount; JdFR
*Marinobacter* env/enrich^39^^,^^g^	–	–	–	–	–	–	−0.4–−0.8°C	Fresh basalt; Arctic oceanic spreading ridges; Norwegian-Greenland Sea

a *Validly published species names as of April 2013*.

b *Carbohydrates used by species are glucose, glycerol, fructose, maltose, mannitol, sucrose, cellobiose, galactose, dextrin, sorbital, trehalose, xylose, ribose, sorbose, erythritol, inositol, dulcitol, arabinose, and N-acetyl-D-glucosamine*.

c–g *87–94%, 89–97%, 96–99%, 99%, 96–98% 16S rRNA gene sequence similarity to Marinobacter species, respectively*.

*Abbreviations: HC, hydrocarbon utilization; CH, carbohydrate utilization; NO^−^_3_, nitrate reduction; gluc ferm, glucose fermentation; AA, amino acid metabolism; Env, environment; Y, yes; N, no; –, unknown; G, genomic evidence; resp, respiratory; st-aer, strict aerobe; env/enrich, environmental/enrichment; JdFR, Juan de Fuca Ridge; TAG, Trans-Atlantic Geotransverse hydrothermal field*.

^1–39^References:

1 *Gauthier et al., 1992*;

2 *Huu et al., 1999 and Márquez and Ventosa, 2005*;

3 *Gorshkova et al., 2003*;

4 *Martín et al., 2003*;

5 *Rontani et al., 2003*;

6 *Shieh et al., 2003 and Validation List no. 94., 2003*;

7 *Yoon et al., 2003*;

8 *Yoon et al., 2004*;

9 *Romanenko et al., 2005*;

10 *Shivaji et al., 2005*;

11 *Takai et al., 2005*;

12 *Green et al., 2006*;

13 *Kim et al., 2006*;

14 *Liebgott et al., 2006*;

15 *Antunes et al., 2007*;

16 *Gu et al., 2007*;

17 *Guo et al., 2007*;

18 *Yoon et al., 2007*;

19 *Xu et al., 2008*;

20 *Montes et al., 2008*;

21 *Zhang et al., 2008*;

22 *Huo et al., 2008*;

23 *Roh et al., 2008*;

24 *Handley et al., 2009a, 2010*;

25 *Wang et al., 2007, 2009*;

26 *Aguilera et al., 2009*;

27 *Zhuang et al., 2009 and Validation List no. 148., 2012*;

28 *Kharroub et al., 2011*;

29 *Qu et al., 2011*;

30 *Kaeppel et al., 2012*;

31 *Liu et al., 2012*;

32 *Lee et al., 2012*;

33 *Sigalevich et al., 2000*;

34 *Wang et al., 2012*;

35 *Edwards et al., 2003*;

36 *Rogers et al., 2003*;

37 *Müller et al., 2010*;

38 *Kaye and Baross, 2000*;

39 Lysnes et al., 2004.

**Table 2:** ***Marinobacter* species attributes**.

***Marinobacter* species**	**Halophilic**	**Optimum salinity (%)**	**Salinity range (%)**	**Mesophilic**	**Optimum temp (°C)**	**Temp range (°C)**	**Optimum pH**	**Major resp. quinone (ubiquinone)**	**G + C (%)^a^**	**Motility mechanism^b^**
*hydrocarbonoclasticus*^1^^,^^2^	s-mod	3–6	0.5–20	Y	32	10–45	7.0–7.5	Q9	(52.7) 57.3	Polar flagellum^c^
*aquaeolei*^2^	slighlty	5	0–20	Y	30	13–50	–	Q9	55.7	Polar flagellum
*excellens*^3^	–	–	1–15	Y	28	10–41	7.5	Q9	56	Polarly flagellated
*lipolyticus*^4^	mod	7.5	1–15	Y	37	15–40	7.5		57	–
*squalenivorans*^5^	–	–	>0	Y	32	–	–		54.3	Polar flagellum
*lutaoensis*^6^	slighlty	3–5	0.5–12	therm	45	25–50	7.0	Q8	63.5	One–several flagella
*litoralis*^7^	s-mod	2–7	0.5–18	psyt	30–37	4–46	7.0–8.5	Q9	55	Polar flagellum
*flavimaris*^8^	s-mod	2–6	>0–20	psyt	37	≤4–45	7.0–8.0	Q9	58	Polar flagellum
*daepoensis*^8^	s-mod	2–6	>0–18	psyt	30–37	>4–45	7.0–8.0	Q9	57	Polar flagellum
*bryozoorum*^9^	–	–	1.0–18	Y	–	7–42	–		59.6	–
*sediminum*^9^	–	–	0.5–18	psyt	–	4–42	–		56.5	–
*maritimus*^10^	slighlty	4	1–13	psyt	22	4–37	8.5	Q9	58	–
*alkaliphilus*^11^	slighlty	2.5–3.5	0–21	Y	30–35	10–45	8.5–9.0	–	57.5	Polar flagellum
*algicola*^12^	s-mod	3–6	1.0–12	psyt	25–30	5–40	7.5	Q9	54–55	Polar flagellum^c^
*koreensis*^13^	s-mod	3–8	0.5–20	Y	28	10–45	6.0–8.0	Q9	54.1	Polar flagellum
*vinifirmus*^14^	s-mod	3–6	0–20	Y	20–30	15–45	6.5–8.4	–	58.7	–
*salsuginis*^15^	slighlty	5	1–20	Y	35–37	10–45	7.5–8.0	Q9	55.9	Polar flagellum
*gudaonensis*^16^	slighlty	2.0–3.0	0–15	Y	–	10–45	7.5–8.0	Q9	57.9	Polar flagellum
*segnicrescens*^17^	s-mod	4–8	1–15	Y	30–37	15–45	7.5–8.0	Q9	62.2	Polar flagellum
*salicampi*^18^	s-mod	8	>0–15	psyt	30	4–39	7.0–8.0	Q9	58.1	Polar flagellum
*pelagius*^19^	slighlty	5.0	0.5–15	psyt	35–30	4–48	7.0–8.0	–	59.0	–
*guineae*^20^	–	–	1–15	psyt	–	4–42	–	Q9	57.1	Polar flagella
*psychrophilus*^21^	–	–	2–8	psyph	16–18	0–22	6.0–9.0	Q9	55.4	–
*mobilis*^22^	slighlty	3.0–5.0	0.5–10.0	Y	30–35	15–42	7.0–7.5	–	58.0–58.9	Polar flagellum
*zhejiangensis*^22^	slighlty	1.0–3.0	0.5–10.0	Y	30–35	15–42	7.0–7.5	–	58.4	Polar flagellum
*goseongensis*^23^	slighlty	4–5	1–25	Y	25–30	10–37	7.5	–	–	–
*santoriniensis*^24^	mod	5–10	0.5–16	Y	35–40	15–45	7–8	Q9	58.1	Polar flagellum
*szutsaonensis*^25^	slighlty	5	0–20	Y	35–40	10–50	7.5–8.0	Q9	56.5	Polar flagellum
*lacisalsi*^26^	mod	7.5	3–15	Y	30–35	20–40	7.0	–	58.6	Polar flagellum
*zhanjiangensis*^27^	slighlty	2–4	1–15	psyt	25–30	4–35	7.5	Q9	60.6	Polar flagellum
*oulmenensis*^28^	mod	5–7.5	1–15	Y	37–40	30–47	6.5–7.0	Q9	57.4	–
*daqiaonensis*^29^	mod	5–10	1–15	Y	30	10–45	7.5	Q9	60.8	Polar flagellum
*adhaerens*^30^	s-mod	2–6	0.5–20	Y	34–38	4–45	7.0–8.5	Q9	56.9	Polar flagellum
*antarcticus*^31^	slighlty	3.0–4.0	0–25	psyt	25	4–35	7.0	–	55.8	Polar flagellum
*xestospongiae*^32^	slighlty	2.0	0.5–6.0	Y	28–36	15–42	7.0–8.0	Q9	57.1	Polar flagellum

a *GC contents range from 54.0–63.5% (average, 57.6%), using the Márquez and Ventosa (2005) value for hydrocarbonoclasticus*.

b *All species are motile, excluding M. goseongensis. M. lutaoensis also has bipolar pili. The number of flagella on M. guineae cells is unknown*.

c *Unsheathed flagellum*.

Abbreviations: Haloph, halophile; Mesoph, mesophile; temp, temperature; resp, respiratory; Y, yes; N, no; –, unknown; s-mod, slightly-moderately; psyt, psychrotolerant; psyph, psychrophile; therm, thermotolerant.

1–32 References: refer to Table 1.

## *Marinobacter hydrocarbonoclasticus*, denitrification and hydrocarbons

The genus was created for *M. hydrocarbonoclasticus*, which was isolated from hydrocarbon-polluted sediment, collected from the mouth of an oil refinery outlet along the French Mediterranean coast (Gauthier et al., 1992), and includes the later heterotypic synonym, *M. aquaeolei* (Huu et al., 1999; Márquez and Ventosa, 2005). The species has an obligate requirement for sodium. It grows readily on complex organic media containing yeast extract and peptone, and aerobically on a range of organic acids (acetate, butyrate, caproate, fumarate, adipate, lactate, citrate), and the amino acids L-glutamate, L-glutamine, and L-proline. Under anaerobic conditions *M. hydrocarbonoclasticus* can perform denitrification using a membrane-bound respiratory NarGHI complex to reduce nitrate (Correia et al., 2008). The nitrite formed is reduced to N_2_ *via* nitrite reductase cytochrome cd1 (Besson et al., 1995), nitric oxide reductase NorBC (EMBL-EBI ABM20188.1, ABM20189.1), and nitrous oxide reductase (Prudêncio et al., 2000). *M. hydrocarbonoclasticus* is most notable, however, for its ability to aerobically degrade liquid and solid, aliphatic (pristane, heneicosane, eicosane, hexadecane, tetradecane) and aromatic (phenanthrene, phenyldecane) hydrocarbons. It uses each hydrocarbon as a sole energy source, and produces large quantities of bioemulsifier. Bioemulsifiers (biosurfactants), are thought to aid in bacterial adhesion to hydrophobic surfaces, water-immiscible material breakdown, and competitor inhibition, and are attracting increasing interest for various industrial applications (Banat et al., 2000; Nerurkar et al., 2009; Williams, 2009; Soberón-Chávez and Maier, 2011).

Of the 34 species named since the genus was created, several exhibit hydrocarbonoclastic activity, while others remain untested (Table 1). Additional hydrocarbons utilized by *Marinobacter* species include squalene, which is metabolized under denitrifying conditions (Rontani et al., 2003), polycyclic aromatic hydrocarbons (PAHs) (Cui et al., 2008), hexane, heptane, petroleum ether (Shivaji et al., 2005; Antunes et al., 2007), *n*-pentadecane, *n*-tridecane, *n*-undecane, *n*-decane, *n*-nonane, butane, and kerosene (Takai et al., 2005).

This hydrocarbonoclastic capacity in *Marinobacter* has attracted attention owing to the potential for these bacteria to remediate crude oil contamination in environments as diverse as the Arabian Gulf (Al-Awadhi et al., 2007) and Artic sea ice (Gerdes et al., 2005). Nitrate reduction by *Marinobacter* species has also been exploited for potential use in oilfield maintenance. Dunsmore et al. (2006) showed reduction of added nitrate prevented deleterious growth of sulfate-reducing bacteria in produced water from a North Sea oilfield oil reservoir, controlling microbial souring reactions. The beneficial reduction of nitrate was largely attributed to indigenous *Marinobacter* species.

## Expanded functional traits of the genus

Following the characterization of *M. hydrocarbonoclasticus*, the functional range of the genus has been further expanded to include (non-exhaustively) fermentation; the ability to respire at least 19 different carbohydrates (Table 1) and several extra amino (e.g., L-alanine, D-glutamate, L-phenylalanine; Antunes et al., 2007 and Green et al., 2006) and organic acids [e.g., malonate, formate, pyruvate, alpha-ketoglutarate; Kim et al. (2006) and Kharroub et al. (2011)]; degradation of the isoprenoid ketone 6,10,14-trimethylpentadecan-2-one (Rontani et al., 1997); growth on ethanol (Gu et al., 2007; Huo et al., 2008), phenol (Liebgott et al., 2006), and various Tweens (e.g., Takai et al., 2005; Green et al., 2006) following enzymatic evidence in *M. hydrocarbonoclasticus*; utilization of fumarate as an electron acceptor (Takai et al., 2005; Handley et al., 2009a); and oxidation/reduction of arsenic, iron or manganese (Handley et al., 2009a,b; Wang et al., 2012).

As for *M. hydrocarbonoclasticus*, all subsequently described species are able to grow aerobically on complex organic matter, and oxidize organic acids. Many, but not all are enzymatically able to reduce nitrate (Table 1). Lack of fermentation by *M. hydrocarbonoclasticus* was initially proposed as a distinctive feature of the genus; however, a number of subsequently isolated type strains exhibit both fermentative and respiratory metabolisms, owing to their ability to ferment glucose (Table 1), lactate (Handley et al., 2009a) and other substrates (Lee et al., 2012). Evaluation of recently available genome sequences also suggests certain *Marinobacter* species may exhibit enzymatic resistance to arsenic and heavy metals (e.g., Wang et al., 2012).

## Biogeography and phylogeny

**Figure 1:**
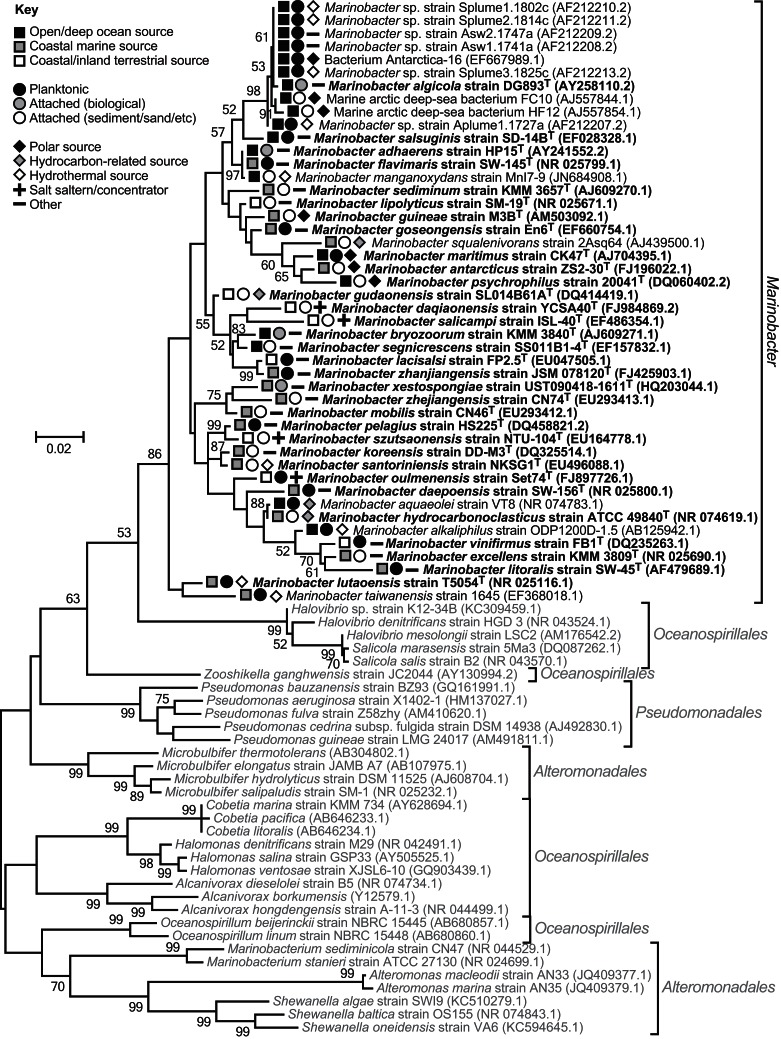
**16S rRNA gene phylogenetic maximum-likelihood tree comparing *Marinobacter* species and their nearest neighbors within the epsilonproteobacterial orders, *Alteromonadales, Pseudomonadales*, and *Oceanospirillales*.** The tree indicates the genus is monophyletic, despite the three orders being non-monophyletic (Williams et al., 2010). The same result was obtained using the neighbor-Joining method. Trees were constructed using MEGA v5.0 (Tamura et al., 2011), Clustal W alignments (Thompson et al., 1994), and 1000 bootstrap replicates. Bootstrap values ≥50 are shown. Sequences used were ≥1350 bp long. *Marinobacter* isolates are in dark font with type species bolded, and closely related *Gammaproteobacteria* are in pale font. GenBank accession numbers are given in parentheses. The symbols indicate *Marinobacter* isolate sources.

*Marinobacter* colonize diverse saline habitats, e.g., sea ice and hydrothermal sediments, facilitated by psychrophilic to thermotolerant physiologies, and an ability to metabolize an array of (in)organic compounds under aerobic or anaerobic conditions. However, evaluation of phylogenetic trees, constructed using 16S rRNA gene sequences, suggest the genus is monophyletic, forming a single clade distinct from other closely related epsilonproteobacterial lineages (Figure 1).

Despite the physiological versatility of the genus, and the ability of some strains to grow (non-optimally) without salt (e.g., Huu et al., 1999; Sigalevich et al., 2000; Liebgott et al., 2006), *Marinobacter* appear to be geographically restricted to marine or terrestrial environments rich in sodium salts. This observation is consistent with the hypothesis that microorganisms exhibit non-random biogeographical differentiation and distribution, due in part to environmental selection (Martiny et al., 2006).

In marine environments, dispersal does not appear to be a limiting factor for *Marinobacter*. Strains have been isolated from, and phylogenetically detected in, oceans (Pacific, Atlantic, Indian, Arctic, and Antarctic) and seas, spanning the globe from pole to equator (Table 1; Kaye et al., 2011). They display both attached and planktonic lifestyles, and the distribution of the genus extends from deep-ocean (hydrothermal) benthic sediment and exposed basalt to surface water, or coastal (hydrothermal) sediment, hot spring water and sand (Table 1; Figure 1).

In many instances, terrestrial isolation sources can be clearly linked to the ocean (e.g., coastal solar salterns and hot springs, and polluted soil from a coastal oil field; Table 1). Isolation of species from terrestrial sources, up to 50–60 km inland, implies a greater degree of terrestrial dispersal (Table 1; Figure 1 and Table S2 in Kaye et al., 2011). However, there is insufficient information regarding the nature of terrestrial isolation sources, and too few isolate and phylogenetic data, to judge how well-dispersed this genus is on land, or whether terrestrial sources are strictly independent from marine influences.

## Lifesytles

In many respects *Marinobacter* species are generalists like their marine and terrestrial *Alteromonadales* cousins in the *Shewanella* genus. *Shewanella* species are respiratory generalists (e.g., Heidelberg et al., 2002), and at least one species (*S. baltica*) has been described as “very close to the ultimate [marine] *r-strategist,”* starkly contrasting with genomically streamlined K-strategist (or oligotrophic) marine bacteria like *Prochlorococcus* (Caro-Quintero et al., 2011). In the presence of surplus organic carbon, *Marinobacter* can grow rapidly, out-competing other bacteria in enrichment cultures (e.g., Handley et al., 2010). This *r*-strategist (or copiotrophic) behavior renders them weed-like and relatively easy to cultivate, even compared with other heterotrophic marine bacteria (Kaye and Baross, 2000). *Marinobacter* can also excel under aerobic-to-anaerobic conditions with no added substrate, while in the presence of Fe(II) (Edwards et al., 2003; Handley et al., 2013a).

This type of lifestyle exhibited by *Marinobacter* strains has been described as opportunistic or “opportunitrophic” (Singer et al., 2011), following the definition given by Moran et al. (2004) in describing the ability of the marine bacterium *Silicibacter pomeroyi* to switch rapid between lithoheterotrophy to heterotrophy in response to nutrient pulses. Use of “-troph” in this context describes the mode of obtaining nourishment (as for the term “psychrotroph”) rather than the source of the nourishment (as in “organotroph”), and as such may be considered a misnomer. The term was applied in order to differentiate between types of fast growing and nominally *r*-strategist bacteria, specifically between specialists (e.g., *Geobacter*, Mahadevan and Lovley, 2008), and generalists like *Marinobacter, Shewanella, Pseudomonas, Vibrio* and *Roseobacter* (Singer et al., 2011)—with the latter two genera already having been deemed opportunistic based on their versatile lifestyles (“opportunitrophs”; Polz et al., 2006). Singer et al. (2011) also identified other potential commonalities shared among the genomes of opportunistic bacteria and *M. aquaeolei* VT8, including a large genomic toolkit for responding to environmental stimuli and for defense (cf. Polz et al., 2006).

There are few phylogenetic studies of the environments from which *Marinobacter* species have been isolated that evaluate their *in situ* relative abundance. Nevertheless, the studies that have been published show two different scenarios for *Marinobacter*. Strains may be characterized as *r*-strategists or opportunistic (Polz et al., 2006; Singer et al., 2011), and dominate communities sporadically when stimulated by high nutrient loads, encountered, for example, in marine aggregates or enrichment cultures (Balzano et al., 2009; Handley et al., 2010). In contrast, relatively high *in situ* abundances of *Marinobacter* (Müller et al., 2010) and uncultured organisms closely related to *Marinobacter* (Rogers et al., 2003; Edwards et al., 2004) have been observed in some hydrothermal systems, implying these organisms may play an important and sustained role in post-depositional mineral alteration.

## Hydrothermal settings

Marine hydrothermal systems are dispersed throughout the world's oceans (Martin et al., 2008), and support abundant psychrophilic-to-mesophilic life even in close proximity to high-temperature venting (Reysenbach and Cady, 2001; Edwards et al., 2003). A number of studies suggest *Marinobacter* may be significant in ‘low-temperature’ hydrothermal systems, defined by low-temperature hydrothermalism (e.g., ~8 to ~40°C, McCollom and Shock, 1997) or ambient seawater temperatures (e.g., ~2° in the deep ocean). This is due to their documented association with several different hydrothermal features (Table 1), and to their ability to heterotrophically or mixotrophically respire inorganic compounds abundant in hydrothermal systems.

A common hydrothermal feature found at plate boundaries, and with which *Marinobacter* or near-relatives have been associated (Edwards et al., 2003; Rogers et al., 2003; Müller et al., 2010), are massive sulfides, which comprise an estimated 6 × 10^8^ tonnes of material globally (Hannington et al., 2011), and adjunct metalliferous sediments. While much of the material for massive sulfides originates from high-temperature hydrothermal fluids (>350°C) emanating from black smoker chimneys (Hannington et al., 2011), particulates distributed locally by plumes and talus from mound and chimney collapse can equilibrate with ambient temperatures (Edwards et al., 2003), or entire mounds can be inactive (Müller et al., 2010). Adjacent to massive sulfide deposits are low-temperature iron- and manganese-rich metalliferous sediments, derived from distal plume fallout with contributions from mound mass wasting (Jannasch and Mottl, 1985; Mills et al., 1993; Hannington et al., 1998)—possibly of the type from which *M. manganoxydans* was isolated (Wang et al., 2012).

Deposits consisting of iron oxyhydroxides, nontronite (a ferric iron-rich clay) and iron-manganese crusts can form independently at plate boundaries or at places of intra-plate volcanism (e.g., Alt, 1988; Karl et al., 1988; Boyd and Scott, 2001; Kennedy et al., 2004; Edwards et al., 2011). They form from diffuse low-temperature venting (Karl et al., 1988; Edwards et al., 2011), and can span areas >100 m^2^ (Boyd and Scott, 2001). Similar deposits exist in shallow marine settings, such as the ferruginous arsenic-rich sediments found in Papua New Guinea and Santorini (Smith and Cronan, 1983; Pichler and Veizer, 1999). *M. santoriniensis* was isolated from the Santorini sediment (Handley et al., 2009a).

Further examples of low-temperature hydrothermal habitats, with which *Marinobacter* or near relatives are associated, include those created by sharp temperature gradients that form across high-temperature chimney walls (Rogers et al., 2003), or buoyant plumes (Kaye and Baross, 2000) that rise 200–300 m up from these vents and spread laterally (German et al., 1991). Exposed, iron-rich basalt, delivered by oceanic spreading centers, provides another environment associated with many deep-sea hydrothermal systems (Lysnes et al., 2004), whereas *M. alkaliphilus* was isolated from alkaline serpentine mud (Takai et al., 2005) from a setting peculiar to mud volcanoes on the non-accretionary Mariana forearc (Fryer et al., 1999).

## Function, biogeochemistry and hydrothermal systems

The various low-temperature hydrothermal settings *Marinobacter*(-like) species inhabit are rich in metals/metalloids, such as iron, manganese, arsenic, copper and zinc (Smith and Cronan, 1983; Hannington et al., 1998) that certain *Marinobacter* strains can transform enzymatically. Moreover, oxygen gradients established in these sediments may be exploited by *Marinobacter* species able to grow heterotrophically under anaerobic/aerobic conditions and mixotrophically under aerobic conditions.

Among the functions *Marinobacter* may perform in these environments is ferrous iron oxidation. The potential for *Marinobacter* Fe(II) oxidation was first suggested by Edwards et al. (2003) after isolating iron-oxidizing bacteria, phylogenetically resembling *M. aquaeolei*, from low-temperature hydrothermal metal sulfides. The isolates were able to grow chemoautotrophically on pyrite, basalt glass and siderite under micro-aerobic conditions. This promoted subsequent study of *M. aquaeolei*, including genome sequencing, and identification of its ability to anaerobically oxidize Fe(II) under mixotrophic conditions (Dhillon et al., 2005; Edwards et al., 2006; Singer et al., 2011). Subsequently, *M. santoriniensis*, which was isolated from iron-rich hydrothermal sediment, was also shown to perform nitrate-dependent Fe(II) oxidation when supplemented with a small amount of organic carbon (Handley et al., 2009a).

Interestingly, *M. santoriniensis* was isolated from sediment rife with stalk-like cells and bacteria phylogenetically resembling iron-oxidizing *Zetaproteobacteria* (Handley et al., 2010). Other *Marinobacter* (or near relatives) were also cultivated from environments containing stalks (Edwards et al., 2003; Lysnes et al., 2004) that speculatively belong to this increasingly characteristic phylum of marine iron-oxidizers—the *Zetaproteobacteria* (Emerson et al., 2007, 2010; Edwards et al., 2011).

As *Marinobacter* are reputedly more versatile than *Mariprofundus ferrooxydans* strains (the sole representatives of the *Zetaproteobacteria*) it is possible they perform other functions in these environments instead of, or in addition to, Fe(II) oxidation. For instance, *Marinobacter* and *Marinobacter*-like isolates have been implicated in Fe(III) reduction, but only in complex or simple co-cultures with other bacteria (Lysnes et al., 2004; Balzano et al., 2009; Handley et al., 2010). This metabolic trait remains to be demonstrated in definitively anexic cultures. *M. santoriniensis* has the genetic potential to reductively detoxify arsenate and mercury using proteins encoded by an *Escherichia coli*-like *arsC* and *merRTA* genes (Handley et al., 2013c), in addition to being able to conserve energy for growth *via* arsenate [As(V)] respiration using an unidentified mechanism, and mixotrophically oxidize arsenite [As(III)] using the *aro* gene cluster—making it one of a handful of bacteria currently known to completely redox-cycle arsenic (Handley et al., 2009b). This is particularly relevant given that the bacterium was isolated from sediment containing ~400 ppm of arsenic.

It remains to be explored whether other *Marinobacter* species share this ability to respire arsenic. However, there is cursory evidence for non-respiratory arsenate reductase (plus/minus putative respiratory arsenite oxidase) genes in several publically available *Marinobacter* genomes (namely, *M. hydrocarbonoclasticus* ATCC49840, GenBank FO203363.1, Grimaud et al., 2012; *M. aquaeolei* VT8, GenBank CP000514.1, Singer et al., 2011; *M. adhaerens* HP15, GenBank CP001978.1, Gärdes et al., 2010; *M. algicola* DG893, GenBank ABCP00000000.1; *Marinobacter* spp. BSs20148 and ELB17, GenBank CP003735.1 and AAXY00000000.1). Likewise, in a recent genome announcement Wang et al. (2012) described a *Marinobacter* candidate, *M. manganoxydans* MnI7-9 that has not only a putative *arsC* gene for arsenic detoxification (GenBank YP_005884959.1), but also a host of other genes that may be used for nickel, mercury, copper, chromate, zinc, cobalt, and cadmium resistance. This bacterium adsorbs and tolerates high levels of metals/metalloids, alongside a demonstrated ability to oxidize manganese, Mn(II), to a mixed-valency Mn(III)/Mn(IV) product *via* an unidentified genetic mechanism. Bacterial manganese oxidation is not thought to be an energy conserving process, but it is considered significant in environmental Mn(IV) oxide formation (Geszvain et al., 2012).

## Conclusions and future directions

Although the genus is widespread in marine settings, and dozens of cultivated representatives and several sequenced genomes exist, the functional breath of *Marinobacter* species remains largely unexplored. The ability to metabolize hydrocarbons and inorganic elements (e.g., iron, arsenic, manganese) has been tested in relatively few species. Information, based on cultures and isolation source characteristics, suggests species within the genus are able to contribute, for example, to the degradation of hydrocarbons in oil-polluted sediment, and the oxidation of Fe(II) in ferruginous sediment or basalt. However, we know little about the nature and magnitude of their actual function in the environment. High-throughput omics (genomics, transcriptomics, proteomics) techniques promise to expand our knowledge into the uncultivated black box that encompasses much of the microbiome, and to facilitate *in situ* investigations of communities (e.g., Ram et al., 2005; Lo et al., 2007; Baker et al., 2012; Handley et al., 2013b), but are limited in part by the large number of genes of unknown function. Much can still be achieved from cultivation experiments. In moving forward, a combination of omics, functional gene expression studies, isotope tracer and cultivation techniques will provide a powerful complement of tools for characterizing both the real and potential function of microorganisms in marine settings and elsewhere, and elucidating the (opportunistic?) role of *Marinobacter* species in environmental biogeochemical cycles.

### Conflict of interest statement

The authors declare that the research was conducted in the absence of any commercial or financial relationships that could be construed as a potential conflict of interest.
